# A Multi-Path MAC Scheduling Scheme for Multi-Channel Wireless Sensor Networks

**DOI:** 10.3390/e25081197

**Published:** 2023-08-11

**Authors:** Fan Zhang, Gangqiang Yang

**Affiliations:** 1 School of Information Engineering, Shandong Management University, Jinan 250357, China; ccfov@126.com; 2 School of Information Science and Engineering, Shandong University, Qingdao 266237, China

**Keywords:** MAC scheduling, random-access, capacity region, computational complexity

## Abstract

Designing reasonable MAC scheduling strategies is an important means to ensure transmission quality in wireless sensor networks (WSNs). When there exist multiple available routes from the source to the destination, it is necessary to combine a data traffic allocation mechanism and design a multi-path MAC scheduling scheme in order to ensure QoS. This paper develops a multi-path resource allocation method for multi-channel wireless sensor networks, which uses random-access technology to complete MAC scheduling and selects the transmission path for each packet according to the probability. Through theoretical analysis and simulation experiments, it can be found that the proposed strategy can provide a reliable throughput capacity region. Meanwhile, due to the use of random-access technology, the computational complexity of the proposed algorithm can be independent of the number of links and channels.

## 1. Introduction

In wireless sensor networks (WSNs), the use of scheduling policies as the MAC layer protocol can bring better QoS than the traditional competition-based CSMA/CD protocol [1]. According to the different methods of resource allocation, one can adopt centralized or distributed methods to achieve network transmission in WSNs. Correspondingly, the MAC scheduling schemes can also be classified as centralized scheduling and distributed scheduling. A centralized scheduler requires the central controller in the network to allocate resources overall, whereas distributed schedulers let each node complete the scheduling and transmission of data messages by itself. In the 5G era, distributed WSNs can serve as the underlying access network below the base station to provide a large number of Internet of Things (IoT) services. Therefore, this paper mainly studies the distributed scheduling strategy in WSNs. As we know, queue-stable is an important state of wireless network systems, which means that the network can bear the current input load [2]. Reasonable MAC scheduling can maintain the queue-stable state of the network system when the input load is located within a certain capacity region. On the contrary, if the network system does not reach a queue state, the queue length may reach infinity, which would impose a huge burden on the network nodes.

**Figure 1:**
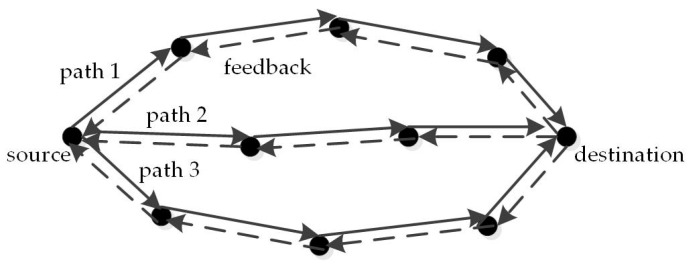
Transmission with multiple routes.

In a single-hop or single-route network scenario, the MAC scheduling algorithm determines whether each link queue transmits packets in the current time slot. In actual network scenarios, if there exist multiple alternative paths available between the source node and the destination node, it is necessary to allocate data traffic (i.e., arriving packets) to different paths reasonably based on MAC scheduling, so as to better balance the network load and obtain better transmission performance [3]. In a multi-path environment, as shown in Figure 1, we aim to ensure a reliable throughput capacity region for a multi-path MAC scheme that combines a distributed MAC scheduler and a data traffic allocation mechanism.

Researchers have made a series of research progress in addressing the distributed MAC scheduling problem. We organize and summarize the current research status from two aspects: single-channel scheduling and multi-channel scheduling and introduce the ideas and innovations of this paper.

### 1.1. Scheduling for Single-Channel Networks

With regard to the distributed MAC scheduling problem in single-channel networks, some researchers have proposed a maximal scheduling (MS) strategy for various interference models [4,5] and demonstrated that MS can ensure a queue-stable state within a certain throughput capacity region. Subsequently, Neely et al. further proposed a multi-path extension scheme for MS [6]. For the purpose of further reducing the implementation complexity of the distributed algorithm, researchers put forward a Q-SCHED (queue-length scheduler) algorithm that completes scheduling by obtaining the queue-length information of neighbors [7,8]. Q-SCHED has been proven to ensure a queue-stable state within a certain throughput capacity region. Moreover, the implementation complexity of Q-SCHED does not increase with the network size. Because of such a feature, Q-SCHED is applicable to large-scale dense networks. In addition, Xie et al. considered the interference intensity and channel gain between links under directional transmission and designed a link scheduling algorithm in directional wireless communication networks [9]. This algorithm utilizes the idea of reinforcement learning to dynamically allocate resources. Each node can independently make intelligent decisions on its own link scheduling by sensing the communication environment within its range, thereby maximizing the network capacity. After analysis, the above-distributed scheduling strategies could all ensure the queue-stable state of the network within a certain throughput capacity region.

### 1.2. Scheduling for Multi-Channel Networks

**Figure 2:**
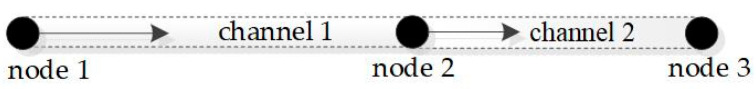
Transmission equipping multiple channels.

In wireless networks, wireless spectrum resources can be divided into multiple different channels, each occupying a certain frequency range. Wireless network nodes can choose a certain channel to transmit data on their own. Thus, links within the same interference area can transmit data without interference by using different channels, which increases throughput and bandwidth [10]. If multiple interfaces are configured for each wireless node, a single node can perform transmission and reception operations simultaneously, which further enhances the transmission efficiency [11], as shown in Figure 2. It should be noted that the application of multiple channels not only improves transmission efficiency and bandwidth but also brings challenges in software implementation.

For distributed MAC scheduling in multi-channel networks, due to capacity differences between different channels, directly extending the single-channel MAC scheduling strategies to a multi-channel scenario may result in extremely poor throughput performance [12,13]. Following this motivation, Lin and Zhang et al. utilized the idea of relay forwarding and developed a distributed SP (single path) algorithm [14] as well as its multi-path extension scheme MP (multiple path) [15]. Yun et al. analyzed the impact of channel switching delay on the performance of scheduling strategies based on SP [16]. Bhandari et al. put forward a MAC scheduling policy without using relay forwarding for multi-channel single-interface networks [17]. Cheng et al. designed an appealing mathematical model. This model can virtualize a multi-interface multi-channel network into a single-channel network. Hence, distributed scheduling strategies in single-channel networks can be directly extended in multi-channel networks without sacrificing throughput performance [18]. Zhang et al. employed the single-channel Q-SCHED scheduling idea to propose a novel distributed algorithm with low complexity (DA-LC) [19,20]. In addition, Xu et al. proposed a low-complexity distributed channel allocation and scheduling strategy (LDCS) [21] in single-interface multi-channel WSNs. The implementation complexity of both DA-LC and LDCS is independent of the network size. However, they can only apply to single-path network environments. In addition to the above achievements, some researchers have systematically analyzed the performance of existing MAC scheduling strategies from the perspectives of throughput and latency [22,23].

### 1.3. Idea and Innovation

In summary, wireless network MAC scheduling technology that ensures QoS has attracted widespread attention, and designing excellent MAC scheduling strategies has become one of the important technical means to solve resource allocation problems in wireless networks. However, by examining existing research results, it can be seen that existing MAC scheduling schemes for multi-path environments still use a maximal-matching mechanism during the scheduling phase. In multi-channel WSNs, such a mechanism brings high implementation complexity. For a multi-channel network containing |*L*| links and |*C*| channels, performing a complete maximal match requires at least *O*(|*L*||*C*|) iterations [24]. Hence, the computational complexity of the multi-path MAC scheduling scheme based on maximal-matching increases with the number of links and channels and is not suitable for large-scale dense distributed networks.

In the 5G era, a large number of IoT applications have emerged. As the underlying network, the network scale of distributed WSNs is becoming larger and the node density is becoming higher. In this case, the implementation complexity of network protocols or algorithms continues to increase, which restricts network performance. It is necessary to reduce the complexity of MAC scheduling strategies in order to meet the requirements of network construction in the 5G era. Based on the above-mentioned analysis, a multi-path MAC scheduling scheme with low complexity, named M-Scheduler, is proposed in this paper. Under the M-Scheduler policy, a tuple-based mathematical model is employed and the random-access technique [25] is used to complete distributed scheduling. Furthermore, a data traffic allocation mechanism is developed. M-Scheduler is proven to ensure the queue-stable state within a reliable throughput capacity region. Meanwhile, a random-access technique is used for scheduling and, hence, the implementation complexity of the algorithm is independent of the network scale. The research results of this paper would provide technical support for the application of MAC scheduling technology in distributed networks in 5G environments.

## 2. System Model

**Figure 3:**
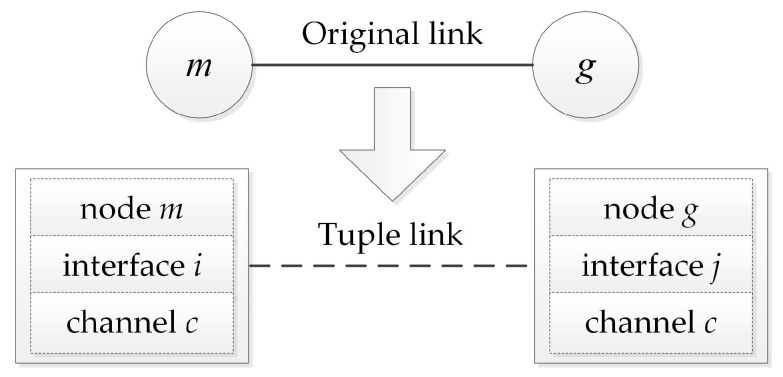
Virtualization process of the tuple-based mathematical model.

Reference [18] points out that using a tuple-based mathematical model can virtualize a multi-interface multi-channel network into a single-channel network. After virtualization, MAC scheduling strategies for single-channel environments can be extended to multi-channel environments, which evidently leads to better performance. Such a tuple-based mathematical model is employed in this paper. Under the tuple-based model, each node, interface, and channel are virtualized as a tuple node. The link between a pair of tuple nodes is defined as a tuple link, as shown in Figure 3. In practical networks, one can use a simple mechanism to implement this model. For example, in a certain time slot, tuple node (*m*, *i*, *c*) works means that the original node *m* works on channel *c* using interface *i*. The tuple link between (*m*, *i*, *c*) and (*g*, *j*, *c*) works means that node *m* uses interface *i* to send data to node *g* on channel *c* and node *g* uses interface *j* to receive data. After virtualization, the set of all tuple links in the network is denoted by *L,* and the set of tuple nodes is *N*. For convenience, in the remaining part of this paper, the word “node” is directly used to represent the tuple node, and “link” represents the tuple link.

**Figure 4:**
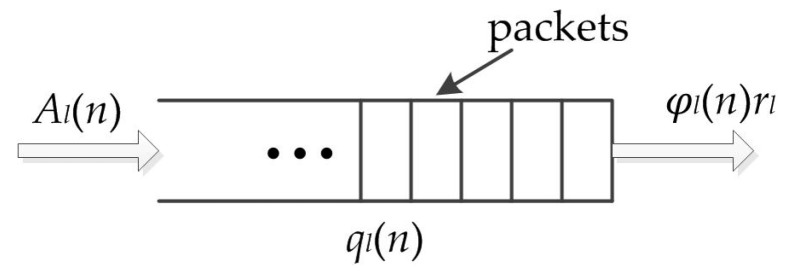
Queue update process.

In the scenario defined in this paper, it is assumed that there are *S* data sources in total and there exist *I*(*s*) transmission paths from each data source node *s* (tuple node) to the destination node. We use *H* as the path indicator variable. If the path *i* of source node *s* passes through link *l*, then we have $H_{si}^{l}=1$; otherwise, $H_{si}^{l}=0$. Assuming that *A_s_*(*n*) represents the number of data packets generated by the source node *s* in time slot *n*. Without loss of generality, it is further assumed that the random variable *A_s_*(*n*) is independent and identically distributed across time slot, and the mean value of *A_s_*(*n*) is *λ_s_*. Hence, the data traffic arriving at link *l* in time slot *n* can be denoted by:

(1) $$A_{l}(n)=\sum_{s=1}^{S}\sum_{i=1}^{I(s)}H_{si}^{l}A_{s}(n)T_{si}(n)$$

where *T_si_*(*n*) represents the proportion of the number of data packets allocated by source node *s* to path *i* in time slot *n*. When a packet arrives in link *l*, it is queued according to the “first in, first out” principle, forming the queue length *q_l_*(*n*) in time slot *n*. Let *r_l_* represent the capacity of link *l* (i.e., transmission rate). According to the output results of the MAC scheduling algorithm, the indicator variable 𝜑*_l_*(*n*) indicates whether link *l* sends data in time slot *n*. If *l* is scheduled and *q_l_*(*n*) > 0, then 𝜑*_l_*(*n*) = 1; otherwise, 𝜑*_l_*(*n*) = 0. Thus, the queue updating is expressed as:

(2) $$\begin{array}{ll} q_{l}(n+1) & ={\left[q_{l}(n)+A_{l}(n)-\varphi_{l}(n)r_{l}\right]}^{+}\\ & ={\left[q_{l}(n)+\sum_{s=1}^{S}\sum_{i=1}^{I(s)}H_{si}^{l}A_{s}(n)T_{si}(n)-\varphi_{l}(n)r_{l}\right]}^{+} \end{array}$$

where [ ]^+^ is the projection on [0, +∞). The queue update process is described in Figure 4.

As we know, there exists interference in the information transmission process in wireless networks. In multi-channel multi-interface networks, interference may come from shared channel or interface conflict, the former is also known as co-frequency interference. The purpose of designing a MAC scheduling strategy is to avoid interference. Based on this motivation, we define an interference set *ε_l_* for each link *l*, which includes all links that may cause interference to *l*. Since we are using a tuple link here, the interference set of *l* includes all cases of channel interference and interface conflicts. Define the interference degree of link *l* as the number of links in the set *ε_l_* that can simultaneously send data. The overall interference degree of a network is defined as the maximum interference degree of all links, represented by the symbol K.

In practical network systems, queue-stable is an important state measuring network throughput performance, which refers to the network being able to bear the current input load. If there exists a MAC scheduling strategy that can maintain the queue-stable state of the network when the input load vector [*λ*_1_, …, *λ_s_*] is located within a certain region Ω, then the region Ω is named as the maximum throughput capacity region. In the multi-path scenario used in this paper, the maximum throughput capacity region Ω can be defined as a set of all input loads [*λ*_1_, …, *λ_s_*] that meet the following conditions:

(3) $$\begin{array}{l} \;\left[\sum_{s=1}^{S}\sum_{i=1}^{I\left(s\right)}H_{si}^{l}\lambda_{s}{\tilde{T}}_{si}\right]\in \Lambda \\ s.t.\sum_{i=1}^{I\left(s\right)}{\tilde{T}}_{si}=1,{\tilde{T}}_{si}\geq 0 \end{array}$$

where the set Λ is the maximum capacity region defined in reference [2] for a single-path network environment. On the other hand, if a MAC scheduling strategy can achieve a capacity region of *k*Ω, then the strategy is called throughput guaranteed, and the efficiency ratio is *k* (0 ≤ *k* ≤ 1). Obviously, a larger value of *k* means that the MAC scheduling strategy has better throughput performance. The multi-path MAC scheduling scheme proposed in this paper is throughput guaranteed. Moreover, the implementation complexity of the proposed scheme is independent of the network size. The following sections will provide a detailed explanation and analysis of the execution process and the performance of the proposed algorithm.

## 3. M-Scheduler Scheme

In multi-path network scenarios, there exist multiple transmission routes between the data source and the destination node. Therefore, in order to ensure the throughput performance of MAC scheduling strategies, it is necessary to simultaneously consider the traffic allocation problem of data packets on multiple paths. Excellent MAC scheduling strategies can always reasonably share the network load and fully utilize the network resources. The detailed implementation process of the multi-path MAC scheduling scheme (named M-Scheduler) proposed in this paper can be divided into the following three steps.

Step 1: Data traffic allocation. In each time slot *n*, when a source node *s* has packets to send, it first calculates the packet allocation proportion vector ***T_s_***(*n*) = [*T_s_*_1_(*n*), …, *T_sI_*_(*s*)_(*n*)]. Such a proportion vector indicates the proportion of data packets sent by the source node to each path and its calculation is completed by solving the following maximum value problem:

(4) $$\begin{array}{ll} \max_{{\vec{T}}_{s}(n)}\; & \left\{-\frac{\phi_{s}}{2}\sum_{i=1}^{I(s)}{\left[T_{si}(n)\right]}^{2}\right.\\ & -\left.\sum_{i=1}^{I(s)}T_{si}(n)\sum_{l=1}^{L}\frac{H_{si}^{l}}{r_{l}}\sum_{j\in \varepsilon_{l}}{\left[\sum_{k\in \varepsilon_{j}}\frac{q_{k}(n)}{r_{k}}\right]}^{\nu }\right\}\\ s.t. & T_{si}(n)\geq 0,\sum_{i=1}^{I(s)}T_{si}(n)=1 \end{array}$$

where *ϕ_s_* and *ν* are positive constants. After solving the problem of (4), the vector ***T_s_***(*n*) can be obtained. In detail, the element in the vector ***T_s_***(*n*) represents the probability of the source node sending a data packet to the corresponding path. For example, if *T_si_*(*n*) = 0.2, the source node *s* sends data to path *i* with a probability of 20% in time slot *n*. In this way, *A_s_*(*n*)*T_si_*(*n*) represents the total number of data packets sent to path *i* by source node *s* in time slot *n*.

**Figure 5:**
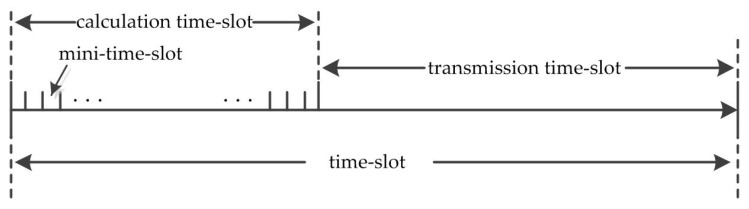
Division of each time slot.

Step 2: Time slot segmentation. In order to make the implementation complexity of the MAC scheduling strategy independent of the network size, it is necessary to divide the time length of each time slot *n* into two parts: a calculation time slot and a transmission time slot, as shown in Figure 5. Furthermore, the calculation time slot is divided into *P* mini time slots. Within time slot *n*, each link selects one of the *P* mini-time-slots according to the mechanism proposed in step 3 as the starting time for data transmission.

Step 3: Scheduling. In each time slot *n*, each link *l* selects a certain mini time slot in the set {1, …, *P* + 1} with probability:

(5) $$\begin{array}{l} \mathrm{Pr}\left\{Y_{l}=P+1\right\}=e^{-\psi_{l}}\\ \mathrm{Pr}\left\{Y_{l}=p\right\}=e^{-\psi_{l}\frac{p-1}{P}}-e^{-\psi_{l}\frac{p}{P}}p=1,\ldots ,P \end{array}$$

where *Y_l_* denotes the mini time slot picked by link *l,* and the parameter *ψ_l_* is calculated using the following formula:

(6) $$\psi_{l}=\alpha \frac{\frac{q_{l}(n)}{r_{l}}}{\max_{j\in \varepsilon_{l}}\left[\sum_{k\in \varepsilon_{j}}\frac{q_{k}(n)}{r_{k}}\right]}$$

where *α* = ln *P*. When link *l* picks *P* + 1, it means that it will not send data in the current time slot. If two neighboring links that interfere with each other (interference may come from a shared channel or interface conflict) choose the same mini time slot, then both will not send data in this time slot. Meanwhile, if link *l* detects other links in its interference set sending data when it is about to start sending, it will remain silent during this time slot. From Formula (6), it can be seen that the calculation of parameter *ψ_l_* requires the neighbor queue information of link *l*, which can be assisted by using the RTS/CTS mechanism in the MAC protocol.

## 4. Performance Analysis

The performance of the M-Scheduler scheme is analyzed here from two aspects: throughput and complexity. With regard to throughput performance, we demonstrate that the proposed M-Scheduler is throughput guaranteed, and the throughput efficiency ratio of the algorithm is further obtained. In terms of complexity, we will explain that the implementation complexity of M-Scheduler is independent of the network size. Based on this characteristic, the M-Scheduler strategy has significant advantages in complexity compared with the existing maximal-matching-based multi-path MAC scheduling schemes.

### 4.1. Throughput Performance

For the purpose of achieving the throughput performance of M-Scheduler, the following lemma is needed.

**Lemma** **1.** *For any positive constant μ > 0, there exists τ_0_ > 0 such that when τ ≥ τ_0_, we have:*

(7) $$\frac{\sum_{j\in L}{\left(\sum_{l\in \varepsilon_{j}}\frac{q_{l}(n)}{r_{l}}\right)}^{1+\tau }}{\underset{j\in L}{\max}\sum_{l\in \varepsilon_{j}}\frac{q_{l}(n)}{r_{l}}}\geq e^{-\mu }\sum_{j\in L}{\left(\sum_{l\in \varepsilon_{j}}\frac{q_{l}(n)}{r_{l}}\right)}^{\tau }.$$

**Proof of Lemma** **1.** Firstly, define a parameter b_j_ regarding link j, denoted as:

(8) $$b_{j}=\frac{\sum_{l\in \varepsilon_{j}}\frac{q_{l}(n)}{r_{l}}}{\underset{u\in L}{\max}\sum_{l\in \varepsilon_{u}}\frac{q_{l}(n)}{r_{l}}}.$$

It can be seen that the value range of *b_j_* is 0 *≤ b_j_ ≤* 1. At the same time, it can be found that there must be a link *j* in the network that makes *b_j_* = 1. Without loss of generality, let *b*_1_ = 1. Hence, if the following inequality holds for every *b_j_* (*j* ∈ *L* and *j* ≠ 1), Lemma 1 is then obtained:

(9) $$1+\sum_{j\in L,j\neq 1}b_{j}{}^{1+\tau }\geq e^{-\mu }\left(1+\sum_{j\in L,j\neq 1}b_{j}{}^{\tau }\right)$$

To prove (9), we divide it into two cases. For any *τ* > 0, if *b_j_* ≥ *e*^−*μ*^, we have (*b_j_*)^1+*τ*^ ≥ (*b_j_*)*^τ^ e*^−*μ*^; if *b_j_ ≤ e^−^^μ^*, then we have (*b_j_*)^1+*τ*^ ≥ 0. Hence, by combining the two cases, the following inequality can be obtained:

(10) $$b_{j}{}^{1+\tau }\geq b_{j}{}^{\tau }e^{-\mu }-e^{-\mu (1+\tau )}.$$

Thus, we further have:

(11) $$1+\sum_{j\in L,j\neq 1}b_{j}{}^{1+\tau }\geq 1+\sum_{j\in L,j\neq 1}b_{j}{}^{\tau }e^{-\mu }-\left(\left|L\right|-1\right)e^{-\mu (1+\tau )}$$

where |*L*| denotes the cardinality of the set *L*. Hence, when the parameter *μ* > 0 is given, we can appropriately choose a positive number *τ*_0_ to make 1 − (|*L*| − 1) *e*^−*μ*(1+^*^τ^*^0)^ ≤ *e*^−*μ*^. Thus, for arbitrary *τ* > *τ*_0_, it is not difficult to deduce (9). Therefore, Lemma 1 was proven. After obtaining Lemma 1, the following theorem is given to describe the throughput performance of M-Scheduler. □

**Theorem** **1.** *Employing the M-Scheduler strategy, for any μ > 0, if the input load vector denoted by **λ** = [λ_1_, …, λ_S_] satisfies*

(12) $$\begin{array}{l} \sum_{l\in \varepsilon_{j}}\frac{\sum_{s=1}^{S}\sum_{i=1}^{I(s)}H_{si}^{l}\lambda_{s}{\tilde{T}}_{si}}{r_{l}}\leq \frac{e^{-\mu }}{1+\mu }\left(1-\frac{\ln P+1}{P}\right)\\ s.t.\sum_{i=1}^{I\left(s\right)}{\tilde{T}}_{si}=1,{\tilde{T}}_{si}\geq 0 \end{array}$$

*then the network system can maintain a queue-stable state.*

**Proof of Theorem** **1.** Firstly, construct the following Lyapunov function:

(13) $$V(n)=\frac{1}{1+\tau }\sum_{j\in L}{\left(\sum_{l\in \varepsilon_{j}}\frac{q_{l}(n)}{r_{l}}\right)}^{1+\tau }$$

To simplify the subsequent proof process, we define two new parameters *x_l_*(*n*) and *Υ_j_* as:

(14) $$\begin{array}{l} x_{l}(n)=E\left[A_{l}(n)\right]=\sum_{s=1}^{S}\sum_{i=1}^{I(s)}H_{si}^{l}\lambda_{s}T_{si}(n)\\ \gamma_{j}=\sum_{l\in \varepsilon_{j}}\frac{q_{l}(n)}{r_{l}} \end{array}$$

where *E*[·] represents the mathematical expectation. Due to *E*[*A_s_*(*n*)*T_si_*(*n*)] = *λ_s_T_si_*(*n*), it can be then obtained that:

(15) $$\begin{array}{l} E\left[V(n+1)-V(n)\left|q(n)\right.\right]\\ \leq \sum_{j\in L}{\left(\gamma_{j}\right)}^{\tau }\left[\sum_{l\in \varepsilon_{j}}\frac{x_{l}(n)}{r_{l}}-\sum_{l\in \varepsilon_{j}}\mathrm{Pr}\left\{Z_{l}\right\}\right]\\ +\sum_{j\in L}o\left[{\left(\gamma_{j}\right)}^{\tau }\right] \end{array}$$

where ***q***(*n*) = [*q_j_*(*n*), *j* ∈ *L*], *Z_l_* indicates the event that link *l* is scheduled, and *o*[·] denotes the higher order infinitesimal. Furthermore, according to Equations (5) and (6), it is not difficult to obtain:

(16) $$\sum_{l\in \varepsilon_{j}}\mathrm{Pr}\left\{Z_{l}\right\}\geq \sum_{p=1}^{P}e^{\left(-\frac{p}{P}\alpha \right)}\left(\sum_{l\in \varepsilon_{j}}\frac{\psi_{l}}{P}\right)\geq \frac{\gamma_{j}}{\underset{k\in L}{\max}\gamma_{k}}\left(1-\frac{\ln P+1}{P}\right)$$

which holds for arbitrary link *j*. Thus, according to Lemma 1, for any positive constant *μ*, there exists *τ*_0_ > 0 such that when *τ* ≥ *τ*_0_, we have:

(17) $$\begin{array}{l} \sum_{j\in L}{\left(\gamma_{j}\right)}^{\tau }\left[\sum_{l\in \varepsilon_{j}}\mathrm{Pr}\left\{Z_{l}\right\}\right]\\ \geq \frac{\left(1-\frac{\ln P+1}{P}\right)}{\underset{k\in L}{\max}\gamma_{k}}\sum_{j\in L}{\left(\gamma_{j}\right)}^{\left(\tau +1\right)}\\ \geq e^{-\mu }\left(1-\frac{\ln P+1}{P}\right)\sum_{j\in L}{\left(\gamma_{j}\right)}^{\tau }. \end{array}$$

Based on the usage conditions of Theorem 1 shown in Equations (12) and (15), we can obtain:

(18) $$\begin{array}{l} E\left[V(n+1)-V(n)\left|q(n)\right.\right]\\ \leq \sum_{j\in L}{\left(\gamma_{j}\right)}^{\tau }\left[\sum_{l\in \varepsilon_{j}}\frac{x_{l}(n)}{r_{l}}-(1+\mu )\sum_{l\in \varepsilon_{j}}\frac{x_{l}^{\#}}{r_{l}}\right]+\sum_{j\in L}o\left[{\left(\gamma_{j}\right)}^{\tau }\right]\\ =\sum_{j\in L}{\left(\gamma_{j}\right)}^{\tau }\left[\sum_{l\in \varepsilon_{j}}\frac{\sum_{s=1}^{S}\sum_{i=1}^{I(s)}H_{si}^{l}\lambda_{s}T_{si}(n)}{r_{l}}\right.\\ \left.\;\;\;\;\;\;-(1+\mu )\sum_{l\in \varepsilon_{j}}\frac{\sum_{s=1}^{S}\sum_{i=1}^{I(s)}H_{si}^{l}\lambda_{s}{\tilde{T}}_{si}}{r_{l}}\right]+\sum_{j\in L}o\left[{\left(\gamma_{j}\right)}^{\tau }\right]\\ =\sum_{s=1}^{S}\lambda_{s}\left[\sum_{i=1}^{I(s)}T_{si}(n)\sum_{l\in L}\frac{H_{si}^{l}}{r_{l}}\sum_{j\in \varepsilon_{l}}{\left(\gamma_{j}\right)}^{\tau }\right.\\ \left.\;\;\;\;\;\;-(1+\mu )\sum_{i=1}^{I(s)}{\tilde{T}}_{si}\sum_{l\in L}\frac{H_{si}^{l}}{r_{l}}\sum_{j\in \varepsilon_{l}}{\left(\gamma_{j}\right)}^{\tau }\right]+\sum_{j\in L}o\left[{\left(\gamma_{j}\right)}^{\tau }\right] \end{array}$$

where $x_{l}^{\#}=\sum_{s=1}^{S}\sum_{i=1}^{I(s)}H_{si}^{l}\lambda_{s}{\tilde{T}}_{si}$. According to the data-traffic-allocation mechanism as in Equation (4), it is not difficult to obtain the following inequality:

(19) $$\begin{array}{l} \sum_{i=1}^{I(s)}\left[T_{si}(n)-{\tilde{T}}_{si}\right]\sum_{l\in L}\frac{H_{si}^{l}}{r_{l}}\sum_{j\in \varepsilon_{l}}{\left(\gamma_{j}\right)}^{\tau }\\ \leq -\frac{\phi_{s}}{2}\sum_{i=1}^{I(s)}{\left[T_{si}(n)\right]}^{2}+\frac{\phi_{s}}{2}\sum_{i=1}^{I(s)}{\left[{\tilde{T}}_{si}\right]}^{2}\\ \leq \phi_{s}\cdot \left[I(s)\right]. \end{array}$$

Hence, based on Equations (18) and (19), we can further deduce that:

(20) $$\begin{array}{l} E\left[V(n+1)-V(n)\left|q(n)\right.\right]\\ \leq -\mu \sum_{j\in L}{\left(\gamma_{j}\right)}^{\tau }\left[\sum_{l\in \varepsilon_{j}}\frac{x_{l}^{\#}}{r_{l}}\right]\\ +\sum_{i=1}^{I(s)}\lambda_{s}\phi_{s}I(s)+\sum_{j\in L}o\left[{\left(\gamma_{j}\right)}^{\tau }\right]. \end{array}$$

Thus, the inequality Equation (20) implies that the network system maintains queue stability [26], and Theorem 1 is proved. □

It should be emphasized that if the interference degree of the entire network is K, then the maximum throughput capacity region Ω can be denoted by the set of all input load vectors ***λ*** meeting:

(21) $$\begin{array}{l} \sum_{l\in \varepsilon_{j}}\frac{\sum_{s=1}^{S}\sum_{i=1}^{I(s)}H_{si}^{l}\lambda_{s}{\tilde{T}}_{si}}{r_{l}}\leq K\\ s.t.\sum_{i=1}^{I\left(s\right)}{\tilde{T}}_{si}=1,{\tilde{T}}_{si}\geq 0 \end{array}$$

for each link *j* [27]. Hence, we can conclude that when the value of *μ* is small enough and *P* is large enough, the right half of inequality Equation (12) approaches 1, i.e.:

(22) $$\lim_{\mu \to 0,P\to +\infty }\frac{e^{-\mu }}{1+\mu }\left(1-\frac{\ln P+1}{P}\right)=1.$$

By comparing Inequalities (12) and (21), it can be concluded that the throughput efficiency ratio of the M-Scheduler strategy is close to 1/K. That is to say, M-Scheduler can ensure the queue-stable state of the network system when ***λ*** ∈ Ω/K. Thus, the proposed scheme is throughput guaranteed. According to the above analysis, the larger the number of mini time slots *P*, the closer the throughput efficiency ratio of M-Scheduler is to 1/K. In the future, the hardware performance of the communication terminal can be improved to ensure that the value of *P* is large enough. In theory, one can compare the throughput performance of M-Scheduler with other existing comparable strategies. The MP algorithm proposed in [15] and the LDCS policy proposed in [21] are both suitable for multi-channel multi-interface network environments with multiple paths and are comparable to the M-Scheduler proposed in this paper. Through comparison, one can find that the efficiency ratio (represented by *ER*) of the three schemes has a relationship of *ER*_M-Scheduler_ > *ER*_LDCS_ > *ER*_MP_. Among them, LDCS adopts a relay-forwarding mechanism in the process of traffic allocation, which brings better throughput performance than the MP algorithm. Furthermore, M-Scheduler utilizes the tuple-based model to virtualize multi-channel networks into single-channel networks, which improves the transmission performance significantly. Therefore, the efficiency ratio of M-Scheduler is greater than that of LDCS. In Section 5, we conduct simulation experiments on the throughput performance of the three schemes to verify the theoretical analysis conclusions.

### 4.2. Implementation Complexity

From the execution process of M-Scheduler, it can be seen that it adopts a distributed implementation mechanism. Compared to centralized implementation, distributed implementation has lower complexity and is easier to deploy in distributed networks such as WSNs. Meanwhile, compared to existing multi-path distributed MAC scheduling schemes applied to wireless multi-channel networks, M-Scheduler does not need to execute the maximal matching processes and does not require iterative operations. The research shows that if the multi-channel network contains |*L*| links and |*C*| channels, the MAC scheduling based on the maximal matching method needs to perform *O*(|*L*||*C*|) iterations in each time slot. Obviously, such implementation complexity is not conducive to the deployment of the corresponding scheduling strategies in large-scale dense wireless networks. Under the proposed M-Scheduler policy, each link selects its own data transmission timestamp based on probability in each time slot. Therefore, the implementation complexity of M-Scheduler does not increase with the number of available channels and links in the network. Hence, it is said that the complexity of M-Scheduler is independent of the network scale and can be better applied to large-scale dense wireless networks.

## 5. Simulations

**Figure 6:**
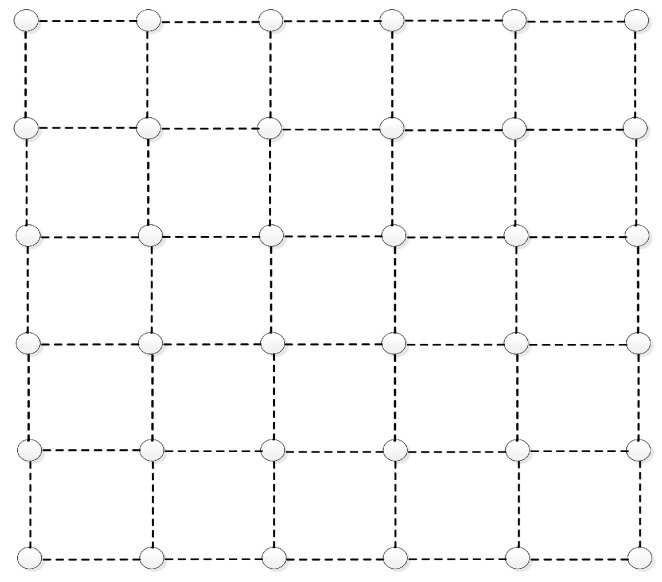
Network topology for simulation.

**Figure 7:**
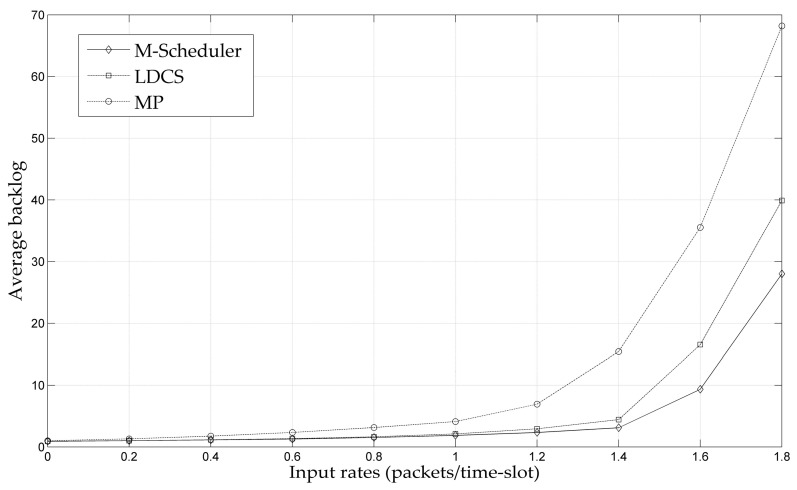
Average backlog versus input rates under scenario 1.

**Figure 8:**
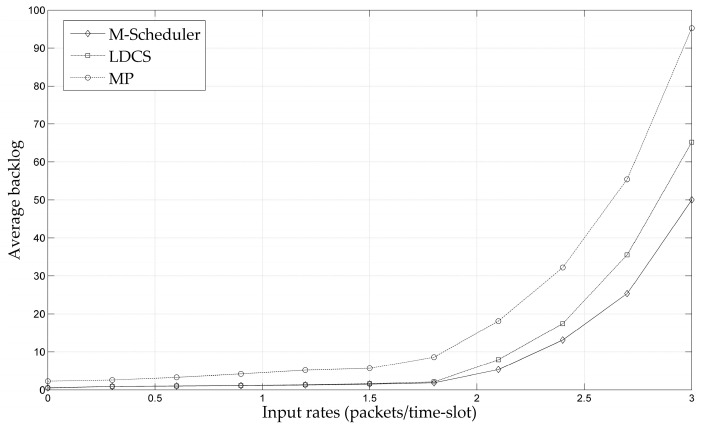
Average backlog versus input rates under scenario 2.

To verify the effectiveness of the multi-path MAC scheduling strategy proposed in this paper in practical network scenarios, the throughput performance of M-Scheduler is compared with the existing MP and LDCS schemes using the NS-2.31 tool. We use a grid topology as shown in Figure 6 with dashed lines representing the original links. A pair of source/destination nodes are selected randomly in the topology. It can be seen that there are multiple alternative paths between the source and the destination. In order to better test the applicability of the algorithm in different network environments, two network scenarios are employed. In scenario 1, there are four available channels with capacities of 2, 2, 1, and 1 packets/time slot, respectively. Each node in the network is configured with 2 wireless interfaces. Under M-Scheduler, the number of divided mini time slots is set to *P* = 10^3^. The simulation results in this scenario are shown in Figure 7. In scenario 2, there are 6 available channels with capacities of 5, 5, 5, 3, 3, and 2 packets/time slots. Configure 3 interfaces for each wireless node and sets the number of mini time slots for the M-Scheduler policy as *P* = 10^5^. The simulation results of scenario 2 are shown in Figure 8.

As shown in Figure 7 and Figure 8, under the three scheduling strategies, when the input load reaches a critical value, the average backlog in the network would sharply increase. Such a critical value of the input load can be considered as the boundary of the throughput capacity region. This phenomenon indicates that the three scheduling strategies are all throughput guaranteed. It can be found that the M-Scheduler proposed in this paper has the largest throughput capacity region among these three algorithms, which is consistent with the theoretical analysis. That is because M-Scheduler virtualizes multi-channel networks into single-channel networks, resulting in improved throughput performance. At the same time, the capacity region of LDCS is larger than that of the MP strategy. The reason is that LDCS adopts a relay-forwarding mechanism to complete data traffic allocation, which can better balance the network load.

Moreover, by comparing Figure 7 and Figure 8, it can be found that the throughput performances of the scheduling strategies in scenario 2 are better than that in scenario 1. That is because there are more available channels with higher transmission rates in scenario 2, which leads to a larger transmission bandwidth. Meanwhile, for the M-Scheduler policy, dividing more mini time slots implies better performance, which is also reflected in the simulation results. The above experiments have confirmed that the M-Scheduler proposed in this paper ensures throughput and can be deployed in practical network systems. In addition to throughput performance, M-Scheduler has significant advantages in terms of complexity, as discussed in detail earlier.

## 6. Conclusions

This paper put forward a novel MAC scheduling scheme for wireless multi-channel networks, named M-Scheduler. The scheme adopts a mathematical model based on tuples, which models nodes, interfaces, and channels as tuple nodes and models the link between a pair of tuple nodes as a tuple link. In this way, a multi-interface multi-channel network can be modeled as a single-channel network. At the same time, when there are multiple transmission paths from the source to the destination in the network, the proposed M-Scheduler first allocates the data traffic to each path reasonably in each time slot and adopts a random-access-based scheduling strategy to complete packet forwarding during the MAC scheduling stage. The theoretical basis for designing a data-traffic-allocation algorithm and random-access probabilities is to ensure the throughput performance of M-Scheduler. By using the Lyapunov analysis method, we have demonstrated that M-Scheduler can achieve a stable throughput capacity region. When the input load is located within the capacity region, the network system maintains a queue-stable state. In addition, due to the utilization of random-access technology to complete MAC scheduling, the implementation complexity of M-Scheduler is independent of the network size. Such a feature makes M-Scheduler perform better in terms of complexity than the existing multi-path MAC scheduling schemes and also enhances its applicability in practical networks.

It should be noted that other types of distributed networks (such as Ad hoc) can also consider using the solution proposed in this paper. The difference lies in the relatively fixed position of nodes in WSNs and, hence, our proposed algorithm is easier to promote and apply in WSNs. In future work, we plan to transplant the M-Scheduler algorithm to the communication protocol stack of WSN terminals and collaborate with IoT companies to conduct practical experiments to verify the effectiveness of M-Scheduler and gradually promote its application in new products.

## Data Availability

No new data were created or analyzed in this study. Data sharing is not applicable to this article.
